# A Computational Model of Dual Competition between the Basal Ganglia and the Cortex

**DOI:** 10.1523/ENEURO.0339-17.2018

**Published:** 2019-01-04

**Authors:** Meropi Topalidou, Daisuke Kase, Thomas Boraud, Nicolas P. Rougier

**Affiliations:** 1 INRIA Bordeaux Sud-Ouest, Talence 33405, France; 2 Institut des Maladies Neurodégénératives, Université de Bordeaux, Bordeaux 33000, France; 3 Institut des Maladies Neurodégénératives, Centre National de la Recherche Scientifique, Unité Mixte de Recherche 5293, Bordeaux 33000, France; 4 Laboratoire Bordelais de Recherche en Informatique, Institut Polytechnique de Bordeaux, Centre National de la Recherche Scientifique, Unité Mixte de Recherche 5800, Université de Bordeaux, Bordeaux 33405, France; 5 Centre National de la Recherche Scientifique, French-Israeli Neuroscience Lab, Bordeaux 33000, France; 6 Centre Hospitalier Universitaire de Bordeaux, Institut MN Clinique, Bordeaux 33000, France

**Keywords:** covert learning, decision making, Hebbian learning, primate, reinforcement learning, theoretical approach

## Abstract

We propose a model that includes interactions between the cortex, the basal ganglia (BG), and the thalamus based on a dual competition. We hypothesize that the striatum, the subthalamic nucleus (STN), the internal globus pallidus (GPi), the thalamus, and the cortex are involved in closed feedback loops through the hyperdirect and direct pathways. These loops support a competition process that results in the ability of BG to make a cognitive decision followed by a motor one. Considering lateral cortical interactions, another competition takes place inside the cortex allowing the latter to make a cognitive and a motor decision. We show how this dual competition endows the model with two regimes. One is driven by reinforcement learning and the other by Hebbian learning. The final decision is made according to a combination of these two mechanisms with a gradual transfer from the former to the latter. We confirmed these theoretical results on primates (*Macaca mulatta*) using a novel paradigm predicted by the model.

## Significance Statement

In this article, we propose a detailed computational model of interaction between basal ganglia (BG) and cortex, in which the former adapts its response according to the outcome while the latter is insensitive to it. The model shows how these two processes interact to issue a unique behavioral answer. This prediction has been verified on monkeys, demonstrating how these two processes are both competing (expression) and cooperating (learning). These results suggest that a behavioral decision emerges actually from a dual competition of two distinct but entangled systems.

## Introduction

Action-outcome (A-O) and stimulus-response (S-R) processes, two forms of instrumental conditioning, are important components of behavior. The former evaluates the benefit of an action to choose the best one among those available (action selection), while the latter is responsible for automatic behavior (routines), eliciting a response as soon as a known stimulus is presented (Mishkin et al., 1984; Graybiel, 2008), independently of the hedonic value of the stimulus. Action selection can be easily characterized by using a simple operant conditioning setup, such as a two-armed bandit task, where an animal must choose between two options of different value, the value being probability, magnitude, or quality of reward (Pasquereau et al., 2007; Guthrie et al., 2013). After some trial and error, a wide variety of vertebrates are able to select the best option (Herrnstein, 1974; Graft et al., 1977; Bradshaw et al., 1979; Matthews and Temple, 1979; Dougan et al., 1985; Herrnstein et al., 1989; Lau and Glimcher, 2005, 2008; Gilbert-Norton et al., 2009). After intensive training, which depends on the species and the task and whether the same values are used throughout the series of the experiments, the animal will tend to become insensitive to change and persist in selecting the formerly best option (Lau and Glimcher, 2005; Yin and Knowlton, 2006). Most of the studies on action selection and habits/routines agree on a slow and incremental transfer from the A-O to the S-R system such that after extensive training, the S-R system takes control of behavior, and the animal becomes insensitive to reward devaluation (Packard and Knowlton, 2002; Seger and Spiering, 2011). Oddly enough, very little is known on the exact mechanism underlying such transfer. One difficult question that immediately arises is when and how the brain switches from a flexible action selection system to a more static one.

Our working hypothesis is that there is no need for such an explicit switch. We propose instead that an action expressed in the motor area results from both the continuous cooperation (acquisition) and competition (expression) of the two systems. To do so, we consider the now classical actor-critic model of decision-making elaborated in the 1980s, which posits that there are two separate components to explicitly represent the policy independently from the value function. The actor is in charge of choosing an action in a given state (policy), while the critic is in charge of evaluating (criticizing) the current state (value function). This classical view has been used extensively for modeling the basal ganglia (BG; Suri and Schultz, 1999; Suri, 2002; Frank, 2004; Doya, 2007; Glimcher, 2011; Doll et al., 2012), although the precise anatomic mapping of these two processes is still subject to debate and may diverge from one model to the other (Redgrave et al., 2008; Niv and Langdon, 2016). However, all these models share the implicit assumption that the actor and the critic are interacting, i.e., the actor determines the policy exclusively from the values estimated by the critic, as in Q-Learning or SARSA. Interestingly enough, Sutton and Barto (1998) noted in their seminal work that one could imagine intermediate architectures in which both an action-value function and an independent policy would be learned.

We support this latter hypothesis based on a decision-making model that is grounded on anatomic and physiologic data and that identify the cortex-BG (CBG) loop as the actor. The critic, of which the substantia nigra pars compacta (SNc) and the ventral tegmental area (VTA) are essential components, interacts through dopamine projections to the striatum (Leblois et al., 2006). Decision is generated by symmetry breaking mechanism that emerges from competitions processes between positives and negatives feedback loop encompassing the full CBG network (Guthrie et al., 2013). This model captured faithfully behavioral, electrophysiological, and pharmacological data we obtained in primates using implicit variant of two-armed bandit tasks that assessed both learning and decision-making, but was less consistent with the explicit version (i.e., when values are known from the beginning of the task) that focus on the decision process only.

We therefore modified this early model by adding a cortical module that has been granted with a competition mechanism and Hebbian learning (Doya, 2000). This improved version of the model predicts that the whole CBG loop is actually necessary for the implicit version of the task; however, when the BG feedback to the cortex is disconnected, the system is nonetheless able to make a decision in the explicit version of the task. Our experimental data fully confirmed this prediction (Piron et al., 2016) and allowed us to solve an old conundrum concerning the pathophysiology of the BG: a lesion or jamming of the output of the BG improve Parkinson patient motor symptoms while it affects marginally their cognitive and psychomotor performances.

An interesting prediction of this generalized actor-critic architecture is that the valuation of options and the behavioral outcome are segregated. In the computational model, it is implied that if we block the output of the BG in a two-armed bandit task before learning, this should induce covert learning during the random choices of the model, because reinforcement learning should still occur at the striatal level under dopaminergic control. The goal of this study is thus two-fold: (1) to present a comprehensive description of the model to provide the framework for an experimental paradigm that allows to disclose covert learning, and (2) to test this prediction in monkeys.

## Materials and Methods

### The task

We consider a variant of a *n*-armed bandit task (Katehakis and Veinott, 1987; Auer et al., 2002) where a player must decide which arm of *n* slot machines to play in a finite sequence of trials to maximize his accumulated reward. This task has received much attention in the literature (e.g., machine learning, psychology, biology, game theory, economics, neuroscience, etc.), because it provides a simple model to explore the trade-off between exploration (trying out a new arm to collect information about its payoff) and exploitation (playing the arm with the highest expected payoff; Robbins, 1952; Gittins, 1979). This task has been shown to be solvable for a large number of different living beings, with a brain (Plowright and Shettleworth, 1990; Keasar, 2002; Steyvers et al., 2009) or without a brain (Reid et al., 2016), and even a clever physical apparatus can solve the task (Naruse et al., 2015).

### The computational task

**Figure 1.**
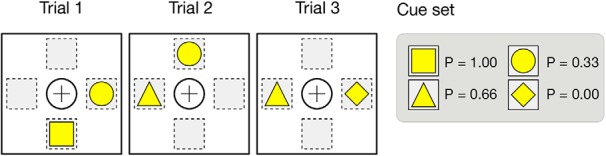
Three task trials from a four-item cue set (☐, ○, Δ, ◊) with respective reward probabilities (1, 0.33, 0.66, and 0).

In the present study, we restrict the *n*-armed bandit task to *n* = 2 with an explicit dissociation between the choice of the option (cognitive choice) and the actual triggering of the option (motor choice). This introduces a supplementary difficulty because only the motor choice, the physical (and visible) expression of the choice, will be taken into account when computing the reward. If cognitive and motor choices are incongruent, only the motor choices matter. Unless specified otherwise, we consider a set of cues {*C_i_*}_i ∈[1,*n*]_ associated with reward probabilities {*P_i_*}_i ∈[1,*n*]_ and a set of four different locations ({*L_i_*}_i ∈[1,4]_) corresponding to the up, down, left, and right positions on the screen. A trial is made of the presentation of two random cues *C_i_* and *C_j_* (*i* ≠ *j*) at two random locations (*L_i_* and *L_j_*) such that we have *L_i_* ≠ *L_j_* (Fig. 1). A session is made of *n* successive trials and can use one to several different cue sets depending on the condition studied (e.g., reversal, devaluation). Unless specified otherwise, in the present study, exactly one cue set is used throughout a whole session.

Once a legal motor decision has been made (i.e., a motor action corresponding to one of the stimulus position), the reward is computed by drawing a random uniform number between 0 and 1. If the number is less or equal to the reward probability of the chosen cue, a reward of 1 is given, otherwise, a reward of 0 is given. If no motor choice has been made or if the motor choice leads to an empty location (illegal choice), the trial is considered to be failed and no reward is given, which is different from giving a reward of 0. The best choice for a trial is defined as the choice of the cue associated with the highest reward probability among the two presented cues. Performance is defined as the ratio of best choices over the total number of trials. A perfect player with full-knowledge can achieve a performance of 1 while the mean expectation of the reward is directly dependent on the cue sampling policy. For example, in Figure 1, if we consider a uniform cue sampling policy for 6*n trials, the mean expected reward for a perfect player with full knowledge is 3/6 × 1 + 2/6 × 2/3 + 1/6 × 1/3 = 14/18 ≈ 0.777…).

### The behavioral task

With kind permission from the authors (Piron et al., 2016), we reproduce here the details of the experimental task which is similar.

**Figure 2.**
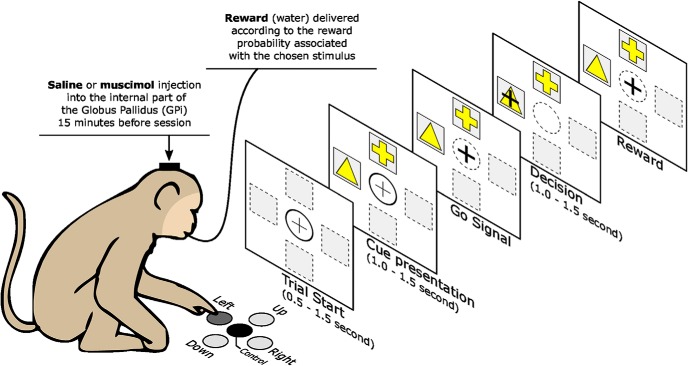
Behavioral task. The monkeys initiate a trial by keeping their hands on the central button, which induced the appearance of the cursor in the central position of the screen. After a random delay, two cues appear in two different positions. The monkey has a random duration time window (0.5–1.5 s) to press the button associated with one cue. It moves the cursor over the chosen cue and has to maintain the position for some duration. After this delay, the monkey is rewarded (0.3 ml of water) or not according to the reward probability of the chosen cue.

The primates were trained daily in the experimental room and familiarized with the setup, which consisted of four buttons placed on a board at different locations (0°, 90°, 180°, and 270°), and a further button in a central position, which detects contact with a monkey’s hand. These buttons correspond to the four possible display positions of a cursor on a vertical screen. The monkeys were seated in chairs in front of this screen at a distance of 50 cm (Fig. 2). The monkeys initiated a trial by keeping their hands on the central button, which induced the appearance of the cursor in the central position of the screen. After a random delay (0.5–1.5 s), two cues appeared in two (of four) different positions determined randomly for each trial. Each cue had a fixed probability of reward (*p*_1_ = 0.75 and *p*_2_ = 0.25) and remains the same during a session. Once the cues were shown, the monkeys had a random duration time window (0.5–1.5 s) to press the button associated with one cue. It moves the cursor over the chosen cue and they have to maintain the position for 0.5–1.5 s. After this delay, the monkeys were rewarded (0.3 ml of water) or not according to the reward probability of the chosen target. The disappearance of the cursor corresponds to an end-of-trial signal, indicating to the monkeys that the trial was finished and they could start a new trial after an intertrial interval between 0.5 and 1.5 s.

### The model

The model is designed to study the implications of a dual competition between the cortex and the BG. It is segregated into three territories partially overlapping at the striatal level (for full discussion, see Guthrie et al., 2013). The motor territory elicits the actual behavioral choice of the model by selecting one of the two positions in which the cues are presented. It roughly corresponds to the supplementary motor area and associated subcortical territories. The cognitive loop chooses one of the two cues that are displayed roughly corresponding to the role devoted to the dorsal lateral prefrontal cortex and associated subterritories. The associative cortex provides a contextual map indicating which cue is presented where on each trial and roughly correspond to the parietal cortex. While in the animal we have access only to the actual choice (provided by the actual behavior of the animal), the model allowed us to have access to the internal choice by looking at which of the two cues was selected at each trial. It could happen that the cognitive loop chooses one cue, while the motor loop chooses the position of the other one, especially at the beginning of the trial, when the synaptic signal-to-noise is still week due to low gain. This cognitive dissonance maybe a mechanism for impulsivity, but it is beyond the scope of this paper.

The competition inside the cortex is conveyed through direct lateral interactions using short-range excitation and long-range inhibition (Wilson and Cowan, 1972, 1973; Coultrip et al., 1992; Deco et al., 2014; Muir and Cook, 2014), while the competition within the BG is conveyed through the direct and hyperdirect pathways (Leblois et al., 2006; Guthrie et al., 2013). Therefore, the indirect pathway and the external segment of the globus pallidus (GPe) are not included. to solve the task, the model relies on the competition between diverging negative feedback loops that provide lateral inhibition, and parallel positive feedback loops that promote differential activation allowing the issue of different cognitive and motor choices. This competitive mechanism occurs at both the basal and cortical level, but the final decision is derived from the cortical level. As soon as the motor cortex activity is above a given threshold, the model is considered to have made a decision. In contrast to (Gurney et al., 2001; Frank, 2004; Doya, 2007), our model relies heavily on feedback mechanisms and closed loops while the latter are purely feed-forward models that merely answer to inputs.

### Architecture

Our model contains five main groups. Three of these groups are excitatory: the cortex, the thalamus, and the subthalamic nucleus (STN). Two populations are inhibitory corresponding to the sensorimotor territories of the striatum and the internal globus pallidus (GPi). The model has been further tailored into three segregated loops (Alexander et al., 1986; Alexander and Crutcher, 1990; Alexander et al., 1991; Mink, 1996; Haber, 2003), namely the motor loop, the associative loop and the cognitive (or limbic) loop. The motor loop comprises the motor cortex (supplementary motor area, primary cortex, premotor cortex, cingulate motor area), the motor striatum (putamen), the motor STN, the motor GPi (motor territory of the pallidum and the substantia nigra), and the motor thalamus (ventrolateral thalamus). The associative loop comprises the associative cortex (dorsolateral prefrontal cortex, the lateral orbitofrontal cortex) and the associative striatum (associative territory of the caudate). The cognitive loop comprises the cognitive cortex (anterior cingulate area, medial orbitofrontal cortex), the cognitive striatum (ventral caudate), the cognitive STN, the cognitive GPi (limbic territory of the pallidum and the substantia nigra), and the cognitive thalamus (ventral anterior thalamus).

### Populations

**Figure 3.**
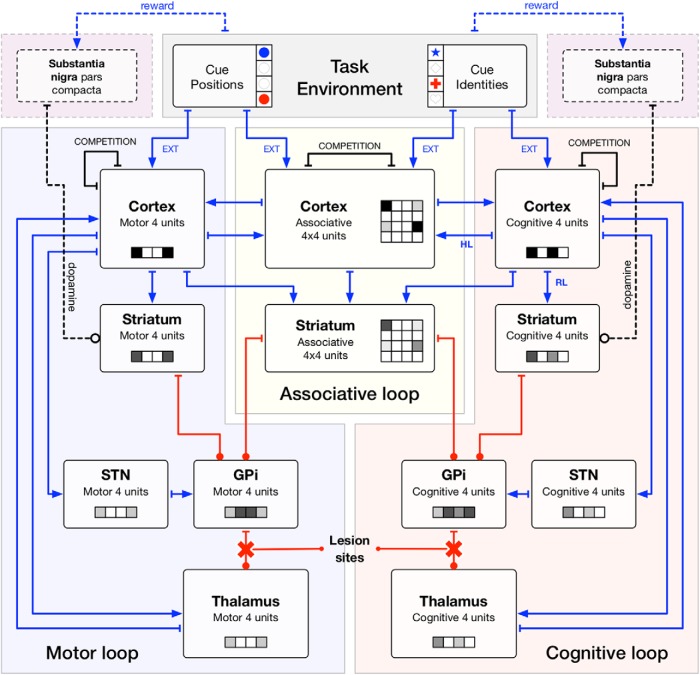
Architecture of the model. The architecture of the model is centered around the hyperdirect pathway (cortex → STN → GPi/SNR → thalamus → cortex), the direct pathway (cortex → striatum→ GPi/SNR → thalamus → cortex) and the cortex where lateral interactions take place. The model is further detailed into three segregated circuits (cognitive, associative, motor). The cognitive and motor circuit each comprises a cortical, a striatal, a thalamic, a subthalamic, and a pallidal population while the associative loop only comprises a cortical and a striatal population. This latter interacts with the two other circuits via diffused connections to the pallidal regions and from all cortical populations. Arrows, excitatory connections. Dots, inhibitory connections.

The model consists of 12 populations: five motor, four cognitive, and two associative populations (Fig. 3). These populations comprise from four to 16 neural assemblies and each possesses a specific geometry whose goal is to facilitate connectivity description. Each assembly is modeled using a neuronal rate model (Hopfield, 1984; Shriki et al., 2003) that give account of the spatial mean firing rate of the neurons composing the assembly. Each assembly is governed by the following equations:

(1) $$\text{τ}\frac{\text{dV}}{\text{dt}}={-\text{V+I}}_{\text{syn}}+I_{ext}+h$$

(2) U=f(V+V.n)

where τ is the assembly time constant (decay of the synaptic input), *V* is the firing rate of the assembly, *I_syn_* is the synaptic input to the assembly, *I_ext_* is the external input representing the sensory visual salience of the cue, *h* is the threshold of the assembly, *f* is the transfer function and *n* is the (correlated, white) noise term. Each population possess its own set of parameters according to the group it belongs to (Table 1). Transfer function for all population but the striatal population is a ramp function [*f*(*x*) = *max*(*x*, 0)]. The striatal population that is silent at rest (Sandstrom and Rebec, 2003), requires concerted coordinated input to cause firing (Wilson and Groves, 1981), and has a sigmoidal transfer function (nonlinear relationship between input current and membrane potential) due to both inward and outward potassium current rectification (Nisenbaum and Wilson, 1995). This is modeled by applying a sigmoidal transfer function to the activation of cortico-striatal inputs in the form of the Boltzmann equation:

$$f(x)=V_{min}+\frac{V_{\max}-V_{\min}}{1+e^{\frac{V_{h}-x}{V_{c}}}}$$

where *V_min_* is the minimum activation, *V_max_* the maximum activation, *V_h_* the half- activation, and *V_c_* the slope. This is similar to the use of the output threshold in the (Gurney et al., 2001) model and results in small or no activation to weak inputs with a rapid rise in activation to a plateau level for stronger inputs. The parameters used for this transfer function are shown in Table 2 and were selected to give a low striatal output with no cortical activation (1 spike/s), starting to rise with a cortical input of 10 spikes/s and a striatal output of 20 spikes/s at a cortical activation of 30 spikes/s.

**Table 1:** Population parameters

Population		Geometry	τ	Threshold	Noise
Cortex	Associative	(4,4)	10 ms	–3	1.0%
	Cognitive	(4,1)	10 ms	–3	1.0%
	Motor	(1,4)	10 ms	–3	1.0%
Striatum	Associative	(4,4)	10 ms	0	0.1%
	Cognitive	(4,1)	10 ms	0	0.1%
	Motor	(4,1)	10 ms	0	0.1%
GPi	Cognitive	(4,1)	10 ms	–10	3.0%
	Motor	(1,4)	10 ms	–10	3.0%
STN	Cognitive	(4,1)	10 ms	–10	0.1%
	Motor	(1,4)	10 ms	–10	0.1%
Thalamus	Cognitive	(4,1)	10 ms	–40	0.1%
	Motor	(1,4)	10 ms	–40	0.1%

**Table 2:** Parameters for striatal sigmoid transfer function

Name	Value
*V_min_*	1
*V_max_*	20
*V_h_*	16
*V_c_*	3

### Connectivity

**Figure 4.**
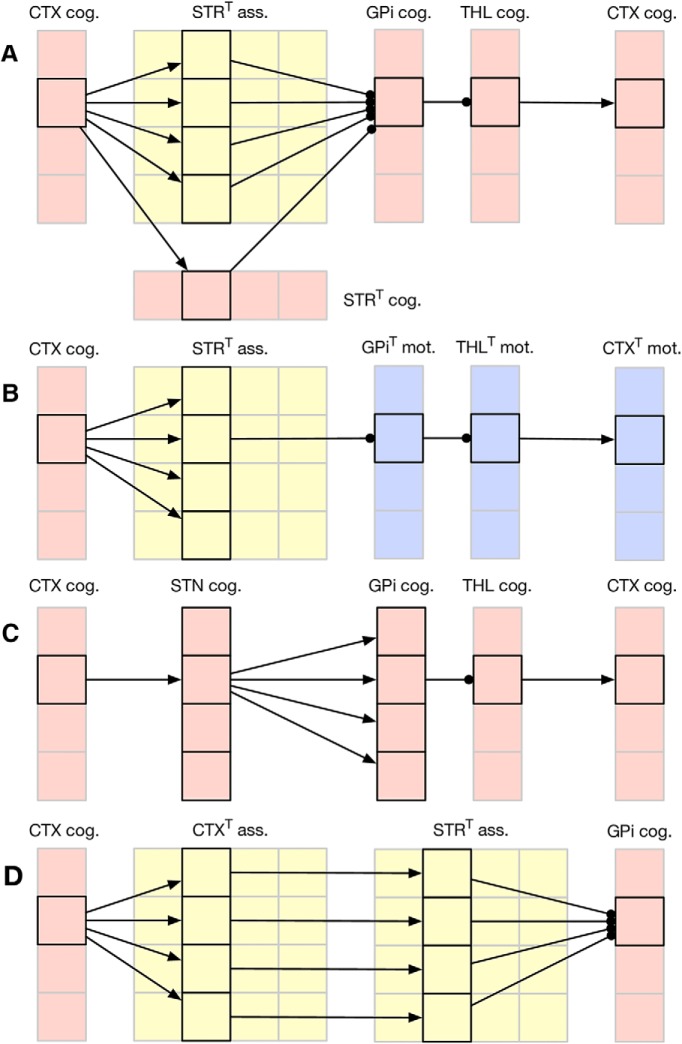
Partial connectivity in the cognitive and associative loops. For clarity, only one assembly has been considered. The motor loop is symmetric to the cognitive one. The T symbol on some name means the geometry of the group has been transposed (for readability). ***A***, The direct pathway from cognitive cortical assemblies diverge from cortex to associative and cognitive striatum. The pathway converges into cognitive GPi, sends parallel projection to the thalamus, and forms a closed loop with the original cognitive cortical assembly. ***B***, Thanks to the convergence of motor and cognitive pathways in associative striatum, there is a cross talk between the motor and cognitive loops. This allows a decision to be made in the cognitive loop to influence the decision in motor loops and vice versa. ***C***, The hyperdirect pathway from cognitive cortical assembly diverges from STN to GPi, innervating all cognitive, but not motor, GPi regions and feeds back to all cognitive cortical assemblies. ***D***, The pathway from associative cortex and associative striatum is made of parallel localized projections.

Although the model takes advantage of segregated loops, they cannot be entirely separated if we want the cognitive and the motor channel to interact. This is the reason why we incorporated a divergence in the cortico-striatal connection followed by a re-convergence within the GPi (Graybiel et al., 1994; Parent et al., 2000; Fig. 4). Furthermore, we considered the somatotopic projection of the pyramidal cortical neurons to the striatum (Webster, 1961) as well as their arborization (Wilson, 1987; Parthasarathy et al., 1992; Cowan and Wilson, 1994; Parent et al., 2000) resulting in specific localized areas of button formation (Kincaid et al., 1998) and small cortical areas innervating the striatum in a discontinuous pattern with areas of denser innervation separated by areas of sparse innervation (Flaherty and Graybiel, 1991; Brown et al., 1998). We also considered the large reduction in the number of neurons from cortex to striatum to GPi (Oorschot, 1996; Bar-Gad and Bergman, 2001). These findings combined lead to striatal areas that are mostly specific for input from one cortical area alongside areas where there is overlap between inputs from two or more cortical areas (Takada et al., 2001) and which are here referred to as the associative striatum.

The gain of the synaptic connection from population *A* (presynaptic) to population *B* (postsynaptic) is denoted as *G*_A→B_, and the total synaptic input to population B is:

$$\begin{array}{c} I_{syn}^{B}=G_{A\to B}\underset{A}{\sum }U_{A} \end{array}$$

where *A* is the presynaptic assembly, *B* is the postsynaptic assembly, and *U_A_* is the output of presynaptic assembly *A*. The gains for each pathway are shown in Table 3. Gains to the corresponding cognitive (motor) assembly are initially five times higher than to each receiving associative area. Reconvergence from cognitive (motor) and association areas of striatum to cognitive (motor) areas of GPi are evenly weighted.

**Table 3:** Connectivity gains and pattern between the different populations

Pop. A	Pop. B	Pathway	Pattern	Gain
Cortex	Striatum	cog. → cog. •	(i,1) → (i,1)	1.0
		mot. → mot.	(i,1) → (i,1)	1.0
		ass. → ass.	(i,j) → (i,j)	1.0
		cog. → ass.	(i,1) → (i,*)	0.2
mot. → ass.(1,i) → (*,i) 0.2				
STN		cog. → cog.	(i,1) → (i,1)	1.0
mot. → mot.(1,i) → (1,i)1.0				
Thalamus		cog. → cog.	(i,1) → (i,1)	0.1
mot. → mot.(1,i) → (1,i)0.1				
Cortex		cog. → cog.	(i,1) → (*,1)	±0.5
		mot. → mot.	(1,i) → (1,*)	±0.5
		ass. → ass.	(i,j) → (*,*)	±0.5
		ass. → mot.	(*,i) → (1,i)	0.025
		ass. → cog.	(i,*) → (i,1)	0.01
		cog. → ass. •	(i,1) → (i,*)	0.025
mot. → ass.(1,i) → (*,i) 0.01				
Striatum	GPi	cog. → cog.	(i,1) → (i,1)	–2.0
		mot. → mot.	(1,i) → (1,i)	–2.0
		ass. → cog.	(i,*) → (i,1)	–2.0
ass. → mot.(*,i) → (1,i)–2.0				
STN	GPi	cog. → cog.	(i,1) → (i,1)	1.0
mot. → mot.(1,i) → (1,i)1.0				
GPi	Thalamus	cog. → cog.	(i,1) → (i,1)	–1.0
mot. → mot.(1,i) → (1,i)–1.0				
Thalamus	Cortex	cog. → cog.	(i,1) → (i,1)	1.0
		mot. → mot.	(1,i) → (1,i)	1.0

For connectivity patterns, * means all. For example, (1,i) → (1,*) means one-to-all connectivity, while (1,i) → (1,i) means one-to-one connectivity. Plastic pathways are indicated by a • symbol.

### Task encoding

**Figure 5.**
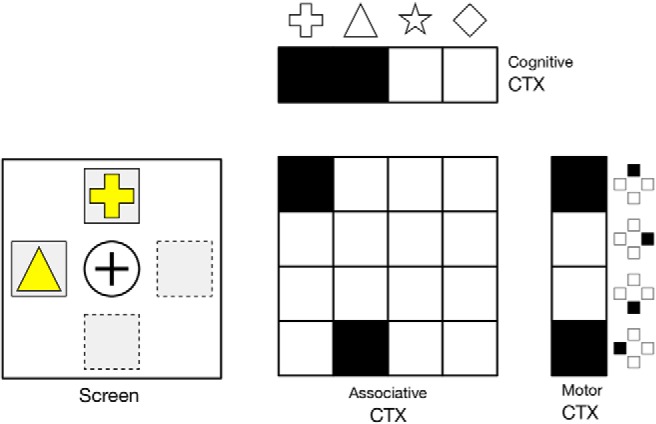
Task encoding. Assemblies in the cognitive cortex encoding the two cues, *C*_1_ and *C*_2_, receive an external current, and assemblies in the motor cortex encoding the two positions, *M*_1_ and *M*_2_, receive a similar external current. These activities are not sufficient to disambiguate between [*C*_1_/*M*_1_, *C*_2_/*M*_2_] or [*C*_1_/*M*_2_, *C*_2_/*M*_1_] (binding problem). This is the reason why the associative cortex encoding one of these two situations receives also an external current, (*C*_1_/*M*_1_, *C*_2_/*M*_2_) to disambiguate the two cases, hence solving the binding problem.

At the trial start, assemblies in the cognitive cortex encoding the two cues, *C*_1_ and *C*_2_, receive an external current (7 Hz) and assemblies in the motor cortex encoding the two positions, *M*_1_ and *M*_2_, receive a similar external current (7 Hz). These activities are ambiguous since they could mean [*C*_1_/*M*_1_, *C*_2_/*M*_2_] or [*C*_1_/*M*_2_,*C*_2_/*M*_1_] (binding problem). This is the reason why the associative cortex encoding one of these two situations receives an external current (7 Hz), (*C*_1_/*M*_1_, *C*_2_/*M*_2_) that allows to bind a stimulus with a position (Fig. 5). The decision of the model is decoded from the activity in the motor cortex only, i.e., independently of the activity in the cognitive cortex. If the model chooses a given cue but produces the wrong motor command, the cognitive choice will not be taken into account, and the final choice will be decoded from the motor command, although that it may lead to an irrelevant choice.

### Dynamics

**Figure 6.**
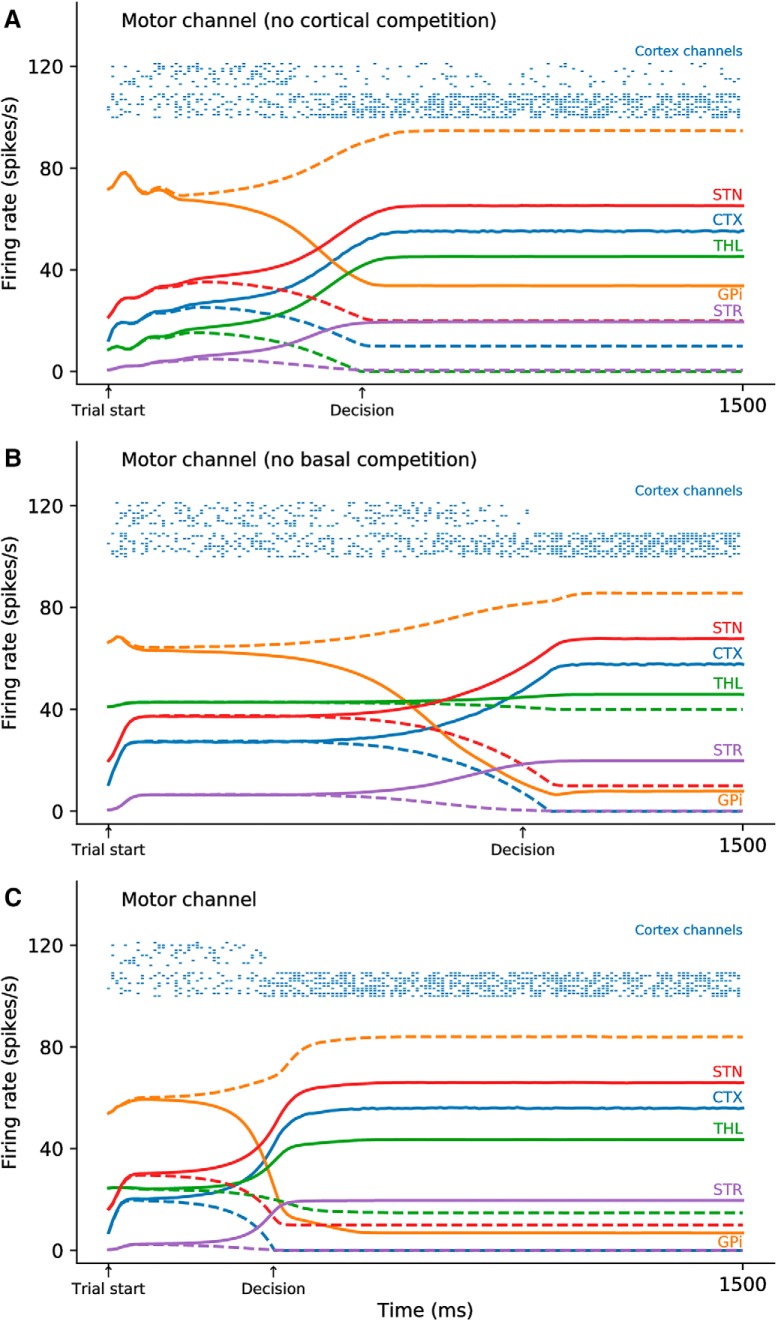
Activity in different populations during a single trial of action selection before learning. The trial starts at time *t* = 0 ms, and the model is allowed to settle to a steady state until the presentation of the cues at *t* = 500 ms. Solid lines represent activity related to the selected population, dashed lines represent activity related to the non-selected population. Decision threshold has been set to 40 spikes/s between the two cortical populations and is indicated on the *x*-axis. Raster plots are related to the cortical populations and has been generated from the firing rate of 10 neurons. ***A***, Activity in the motor populations in the absence of lateral competition in the cortical populations. The damped oscillations during the settling phase are characteristic of the delayed feedback from the STN (excitation) and the striatum (inhibitory) through the globus pallidus and the thalamus. ***B***, Activity in the motor populations in the absence of the feedback from the BG (GPi) to the cortical populations via the thalamus. Decision threshold is reached thanks to the direct lateral competition in both cognitive and motor cortical channels. There is no damped oscillation, since there is no delay between the cortical populations, and the decision times are slower than in the previous case. ***C***, Activity in the motor populations in the full model with a dual competition, one cortical and one basal. When congruent (cortical and basal decision are the same), decision time for both the motor and cortical channels are faster than in the absence of one of the competition loop.

Two different competition mechanisms exist inside the model. One is conveyed through the direct and hyperdirect pathways, the other is conveyed inside the cortex through short-range excitation and long-range inhibition. The former has been fully described and analyzed in Leblois et al. (2006), while the latter been extensively studied in a number of experimental and theoretical papers (Wilson and Cowan, 1972, 1973; von der Malsburg, 1973; Amari, 1977; Callaway, 1998; Taylor, 1999). Each of these two competition mechanisms can lead to a decision as illustrated in Figure 6, which shows the dynamic of the motor loop for all the population in three conditions. In the absence of the cortical interactions (gain of cortical lateral connections has been set to 0), the direct and hyperdirect pathway are able to promote a competition that results in the selection of one of the two assemblies in each group. In the absence of GPi output (connection has been cut), the cortical lateral connections are able to support a competition resulting in the selection of one of the two assemblies, although such decision is generally slower than decisions formed in the BG. The result of the dual competition is a faster selection of one of the two assemblies after learning, when there is no possibility for the two competitions to be non-congruent (one competition tends to select move A while the others tend to select move B). We will see in the results section that if the result of the two competitions is non-congruent, the decision is slower.

### Learning

Learning has been restricted to the cognitive channel on the cortico-striatal synapse (between the cognitive cortex and striatum) and the corticocortical synapses (between the cognitive and associative cortex). Most probably there is learning in other structures and pathways, but the aim here is to show that the proposed restriction is sufficient to produce the behavior under consideration. All synaptic weights are initialized to 0.5 (SD, 0.005) and used as a multiplier to the pathway gain to keep the factors of gain and weight separately observable. All weights are bound between *W*min and *W*max (Table 4) such that for any change Δ*W* (*t*), weight *W* (*t*) is updated according to the equation:

$$W(t)\leftarrow W(t)+\Delta W(t)(W_{\text{max}}-W(t))(W(t)-W_{\text{min}})$$

**Table 4:** Learning parameters

Name	Value
*W_min_*	0.25
*W_max_*	0.75
*LTP_RL_*	0.050
*LTD_RL_*	0.030
*LTP_HL_*	0.005
α	0.025

### Reinforcement learning

At the level of cortico-striatal synapses, phasic changes in dopamine concentration have been shown to be necessary for the production of long-term potentiation (LTP; Kerr and Wickens, 2001; Reynolds et al., 2001; Surmeier et al., 2007; Pawlak and Kerr, 2008). After each trial, once reward has been received (0 or 1), the cortico-striatal weights are updated according to the reward prediction error (RPE):

(3) $${\Delta W}_{B}^{A}=LTP_{\text{RL}}\times RPE\times U_{B}\,\;\mathrm{if}\;\mathrm{RPE}>0$$

(4) $$=\;LTD_{\text{RL}}\times RPE\times U_{\text{B}}\;\mathrm{if}\;\mathrm{RPE}<0$$

where ${\Delta W}_{B}^{A}$ is the change in the weight of the cortico-striatal synapse from cortical assembly A to striatal assembly B, *RPE* is the RPE, the amount by which the actual reward delivered differs from the expected reward, *U*B is the activation of the striatal assembly, and α is the actor learning rate. Generation of LTP and long-term depression (LTD) in striatal MSNs has been found to be asymmetric (Pawlak and Kerr, 2008). Therefore, in the model, the actor learning rate is different for LTP and LTD. The RPE is calculated using a simple critic learning algorithm:

$$RPE=R-V_{\text{i}}$$

where R, the reward, is 0 or 1, depending on whether a reward was given or not on that trial. Whether a reward was given, it was based on the reward probability of the selected cue (which is the one associated with the direction that was chosen); i is the number of the chosen cue, and *V_i_* is the value of cue i. The value of the chosen cue is then updated using the RPE:

$$V_{\text{i}}\leftarrow V_{\text{i}}+\alpha RPE$$

### Hebbian learning

At the level of corticocortical synapses, only the co-activation of two assemblies is necessary for the production of LTP (Bear and Malenka, 1994; Caporale and Dan, 2008; Feldman, 2009; Hiratani and Fukai, 2016). After each trial, once a move has been initiated, the corticocortical weights are updated according to:

$$\Delta W_{B}^{\text{A}}=LTP_{\text{HL}}\;\times \;U_{\text{A}}\times U_{\text{B}}$$

where ${\Delta W}_{B}^{A}$ is the change in the weight of the corticocortical synapse from cognitive cortical assembly A to associative cortical assembly B. This learning rule is thus independent of reward.

### Experimental setup

Experimental data were obtained from two female macaque monkeys (*Macaca mulatta*). Experiments were performed during the daytime. Monkeys were living under a 12/12 h light/dark diurnal rhythm. Although food access was available *ad libitum*, the primates were kept under water restriction to increase their motivation to work. A veterinary skilled in health care and maintenance in nonhuman primates supervised all aspects of animal care. Experimental procedures were performed in accordance with the Council Directive of 20 October 2010 (2010/63/UE) of the European Community. This project was approved by the French Ethic Committee for Animal Experimentation (50120111-A).

### Surgical procedure

Cannula guides were implanted into the left and right GPi in both animals under general anesthesia. Implantation was performed inside a stereotaxic frame guided by ventriculography and single-unit electrophysiological recordings. A ventriculographic cannula was introduced into the anterior horn of the lateral ventricle and a contrast medium was injected. Corrections in the position of the GPi were performed according to the line between the anterior commissure (AC) and the posterior commissure (PC) line. The theoretical target was AP: 23.0 mm, L: 7.0 mm, P: 21.2 mm. A linear 16-channel multielectrode array was lowered vertically into the brain. Extracellular single-unit activity was recorded from 0 to 24 mm relative to the AC–PC line with a wireless recording system. Penetration of the electrode array into the GPi was characterized by an increase in the background activity with the appearance of active neurons with a tonic firing rate (around the AC–PC line). The exit of the electrode tips from the GPi was characterized by the absence of spike (around 3–4 mm below the AC–PC line). When a clear GPi signal from at least three contacts had been obtained, control radiography of the position of the recording electrode was performed and compared to the expected position of the target according to the ventriculography. If the deviation from the expected target was less than 1mm, the electrode was removed and a cannula guide was inserted with a spare cannula inside so that the tip of the cannula was superimposed on the location of the electrode array in the control radiography. Once the cannula guide was satisfactorily placed, it was fixed to the skull with dental cement.

### Bilateral inactivation of the GPi

Microinjections were delivered bilaterally 15 min before a session. For both animals, injections of the G AB AA agonist muscimol hydrobromide (Sigma) or saline (NaCl 9) were randomly assigned each day. Muscimol was delivered at a concentration of 1 µg/µl (dissolved in a NaCl vehicle). Injections (1 µl in each side) were performed at a constant flow rate of 0.2 µl/min using a microinjection system. Injections were made through a 30-gauge cannula inserted into the two guide cannula targeting left and right GPi. Cannulas were connected to a 25-µl Hamilton syringe by polyethylene cannula tubing.

### Data analysis

Theoretical and experimental data were analyzed using Kruskal-Wallis rank sum test between the three conditions [saline (C0), muscimol (C1) or saline following muscimol (C2)] for the six samples [12 × 10 first trials of C0 (control), 12 × 10 last trials of C0 (control), 12 × 10 first trials of C1 (GPi Off/muscimol), 12 × 10 last trials of C1(GPi Off/muscimol), 12 × 10 first trails of C2(GPi On/saline), 12 × 10 last trials of C2(GPi On/saline)] with *post hoc* pairwise comparisons using Dunn’s test for multiple comparisons of independent samples; *p* values have been adjusted according to the false discovery rate (FDR) procedure of Benjamini–Hochberg. Results were obtained from raw data using the PMCMR R package (Pohlert, 2014). Significance level was set at *p* < 0.01. Experimental raw data is available from (Kase & Boraud, 2017) under a CC0 license, theoretical raw data and code are available from (Rougier & Topalidou, 2017) under a CC0 license (data) and BSD license (code). The data and the codes are also available as extended data (respectively model codes and experimental data files). 

## Results

Our model predicts that the evaluation of options and the behavioral outcome are two separate (but entangled) processes. This means that if we block the output of the BG before learning, reinforcement learning still occurs at the striatal level under dopaminergic control and should induce covert learning of stimuli value although the behavioral choice would appear as random.

### Protocol

The protocol has been consequently split over two consecutive conditions (C1 and C2) using the same set of stimuli and a dissociated control (C0) using a different set of stimuli (using same probabilities as for C1 and C2):

C0 60 trials, GPi On (model), saline injection (primates), stimulus set 1 (*A*_1_, *B*_1_) with *PA*_1_ = 0.75, *PB*_1_ = 0.25

C1 60 trials, GPi Off (model), muscimol injection (primates), stimulus set 2 (*A*_2_, *B*_2_) with *PA*_2_ = 0.75, *PB*_2_ = 0.25

C2 60 trials, GPi On (model), saline injection (primates), stimulus set 2 (*A*_2_, *B*_2_) with *PA*_2_ = 0.75, *PB*_2_ = 0.25

### Computational results

**Figure 7.**
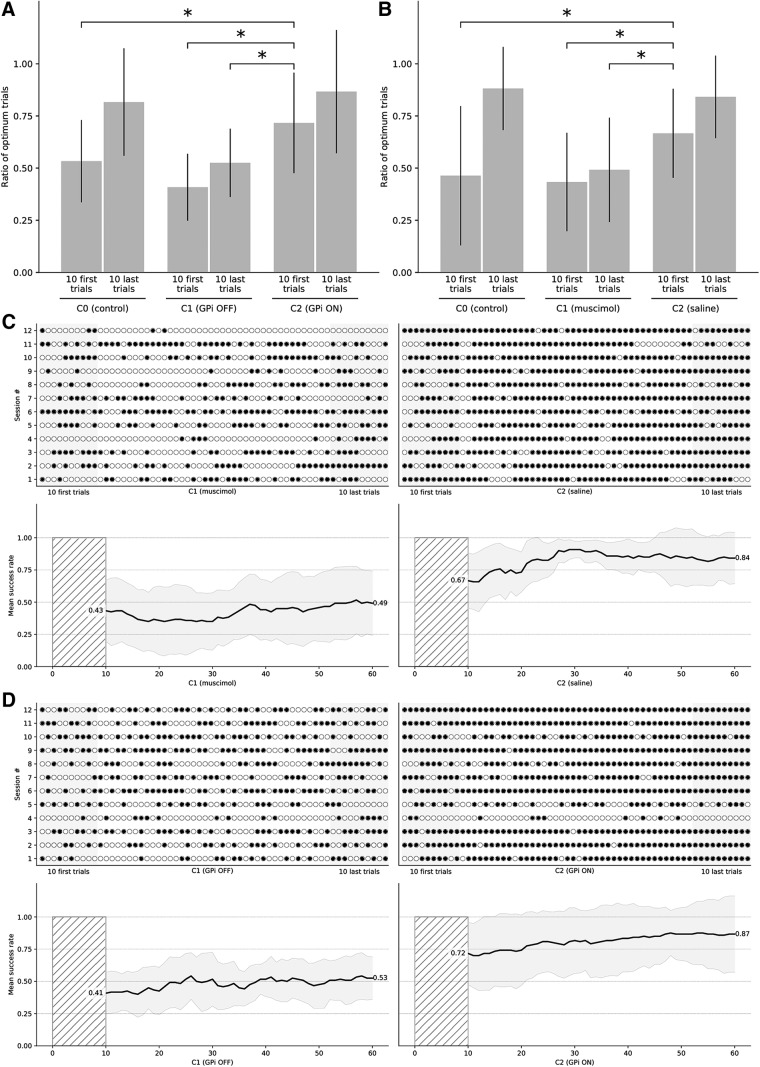
Theoretical and experimental results. Histograms show the mean performance at the start and the end of a session in C1 and C2 conditions for both the model (***A***) and the monkeys (***B***). At the start of C2, the performance for both the model and the monkeys is significantly higher compared to the start and end of C1, suggesting that covert learning has occurred during C1 although performances are random during C1. ***C***, Individual trials (*n* = 2 × 60) for all the sessions (*n* = 12) for the primates (monkey 1: sessions 1–7, monkey 2: sessions 8–12). ***D***, Individual trials (*n* = 2 × 60) for all the sessions (*n* = 12) for the model. A black dot means a successful trial (the best stimulus has been chosen), an outlined white dot means a failed trial (the best stimulus has not been chosen). Measure of success is independent of the actual reward received after having chosen one of the two stimuli. The bottom part of each panel shows the mean success rate over a sliding window of ten consecutive trials and averaged across all the sessions. The thick black line is the actual mean and the gray-shaded area represents the SD over sessions.

We tested our hypothesis on the model using 12 different sessions (corresponding to 12 different initializations of the model). On day 1 (condition C1), we suppressed the GPi output by cutting the connections between the GPi and the thalamus. When the GPi output has been suppressed, the performance is random at the beginning, as shown by the average probability of choosing the best option (expressed as mean ± SD) in the first 10 trials (0.408 ± 0.161), and remains so until the end of the session (0.525 ± 0.164). Statistical analysis revealed that no significant difference between the 10 first and 10 last trials. On day 2 (condition C2), we reestablished the connections between GPi and thalamus and tested the model to the same task as in C1 using the same set of stimuli. Results show a significant change in behavior: the model starts with an above-chance performance on the first 10 trials (0.717 ± 0.241), and this change is significant (Table 5; Fig. 7) compared to the start of C1, compared to the end of C1 and compared to the start of C0, confirming our hypothesis that the BG have previously learned the value of stimuli although they were unable to alter the behavior of the model.

**Table 5:** Theoretical results statistical analysis on correct answers

**H0**	**statistic (H)**	**p value**
C0 start = C2 start	2.965	0.0051
C1 start = C2 start	4.986	1.8e-6
C1 end = C2 start	3.099	0.0036

Kruskal–Wallis rank sum test between the three conditions [saline (C0), muscimol (C1), or saline following muscimol (C2)] with *post hoc* pairwise comparisons using Dunn’s test for multiple comparisons of independent samples. The script used for the analysis (R language) is available from Rougier and Topalidou (2017).

### Experimental results

We tested the prediction of the model on two female macaque monkeys which have been implanted with two cannula guides into their left and right GPi (for details, see Materials and Methods). To inhibit the GPi, we injected bilaterally a GABA agonist (muscimol, 1µg) 15 min before working session on day 1 (condition C1). The two monkeys were trained for seven and five sessions, respectively, using the same set of stimuli for each session. Results show that animals were unable to choose the best stimulus in such condition from the start (0.433 ± 0.236) to the end (0.492 ± 0.250) of the session. Statistical analysis revealed no significant difference between the 10 first and 10 last trials in C1. On day 2 (condition C2), we injected bilaterally a saline solution 15 min before working session, and animals had to perform the same protocol as in C1. Results show a significant change in behavior (Table 6; Fig. 7): animals start with an above-chance performance on the first 10 trials (*p* = 0.667 ± 0.213), compared to the start of C1, compared to the end of C1 and compared to the start of C0, confirming our hypothesis that the BG has previously learned the value of stimuli.

**Table 6:** Experimental results

**H0**	**statistic (H)**	**p value**
C0 start = C2 start	3.181	0.0024
C1 start = C2 start	3.738	0.0004
C1 end = C2 start	2.803	0.0069

Statistical analysis on correct answers. Kruskal–Wallis rank sum test between the three conditions [saline (C0), muscimol (C1), or saline following muscimol (C2)] with *post hoc* pairwise comparisons using Dunn’s test for multiple comparisons of independent samples. The script used for the analysis (R language) is available from Rougier and Topalidou (2017).

## Discussion

### Revisiting an old idea

The model architecture we proposed in this manuscript is not totally original in the sense that the model implements known pathways that have been established for quite a long time and taken into account in a number of models. More precisely, several computational models in the literature include both the inner BG pathways as well as the feed-forward and feed-back loops from and to the cortex (through thalamus). However, most of these models (if not all) put a specific emphasis on the role of the BG without considering the cortex as a decision-making structure. To the best of our knowledge, virtually none of these models take advantage of a dual competition mechanism similar to the one we introduced. For example, the model by O’Reilly and Frank (2006), which solves the temporal and structural credit assignment problems on a working memory task, includes a Hebbian learning component for the posterior cortical part; however, O’Reilly and Frank (2006) show that Hebbian learning is not critical for performances (only a 5% drop in performance) and did not specifically study lesions in the BG. Similarly, the model by Brown et al. (2004) does include a laminar frontal part with a specific emphasis on the interaction between the BG and the frontal cortex and explain how to balance between reactive and planned behaviors. However, authors considers that “lesions of the BG uniquely cause devastating disorders of the voluntary movement system,” which is not always the case as we have shown with experimental data (Desmurget and Turner, 2010; Piron et al., 2016). The model by Schroll et al. (2014) and Villagrasa et al. (2018) is notably similar to our own model and suggests that the CBG pathway is not required to perform previously well-learned SR associations, which is quite consistent with our own hypothesis. By using a simple S-R association tasks, authors show that a focal GPi lesion do not impact significantly performances over a previously well learned task. This is made possible thanks to the cortico-thalamic pathway that learn “to interconnect those cortical and thalamic neurons that are simultaneously activated via reward-sensitive BG pathways.” The main difference with our own model is the localization of the Hebbian learning and the lateral competition. We hypothesize this learning to occur at the cortical level and take advantage of a lateral competition mechanism that is necessary to solve our decision task (while it is not necessary for a simple S-R task). This lateral competition acts indeed as a Go/NoGo substitute in the absence of the BG output. Furthermore, authors did not specifically conclude on the presence of covert learning when GPi is lesioned. They showed that the model has very bad performance when learning a new task, but they did not test the model once GPi is unlesioned. We suspect that if they had tested it, they would have found results similar to our own.

### Covert learning in the BG

These results reinforce the classical idea that the BG architecture is based on an actor critic architecture where the dopamine serves as a reinforcement signal. However, the proposed model goes beyond this classical hypothesis and proposes a more general view on the role of the BG in behavior and their entanglement with the cortex. Our results, both theoretical and experimental, suggest that the critic part of the BG extends its role beyond the BG and makes it *de facto* a central component in behavior that evaluates any action, independently of their origin. This hypothesis is very congruent with the results introduced in Charlesworth et al. (2012), where authors show that the anterior forebrain pathway in Bengalese finches contributes to skill learning even when it is blocked and does not participate in the behavioral performance. This is also quite compatible with the hypothesis that the BG is a general purpose trainer for corticocortical connections as proposed by Ashby et al. (2010) and Hélie et al. (2015). Here, we introduced a precise computational model using both reinforcement and Hebbian learning, supported by experimental data, that explains precisely how this general purpose trainer can be biologically implemented.

**Figure 8.**
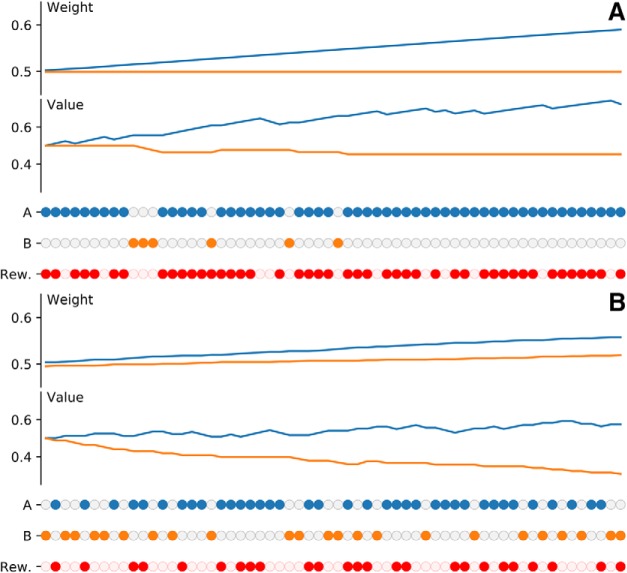
Model performance during a single session. Filled dots indicate the chosen cue between A and B. Filled red dots indicate if a reward has been received following the choice. Reward probability is 0.75 for cue A and 0.25 for cue B, but the displayed values are computed according to the actual reward received for each option. They are based on the history of the session, not the theoretical values. ***A***, Intact model (C0). The BG output drives the decision and evaluates the value of cue A and cue B with a strong bias in favor of A, because this cue is chosen more frequently. In the meantime, the Hebbian weight relative to this cue is strongly increased, while the weight relative to the other cue does not change significantly. ***B***, Lesioned model (C1). The BG output has been suppressed and decisions are random. Hebbian weights for cue A and cue B are both increased up to similar values at the end of the session. In the meantime, the value of cue A and cue B are evaluated within the BG and the random sampling of cue A and cue B leads to an actual better sampling of value A and B. This is clearly indicated by the estimated value of B that is very close to the theoretical value (0.25).

**Figure 9.**
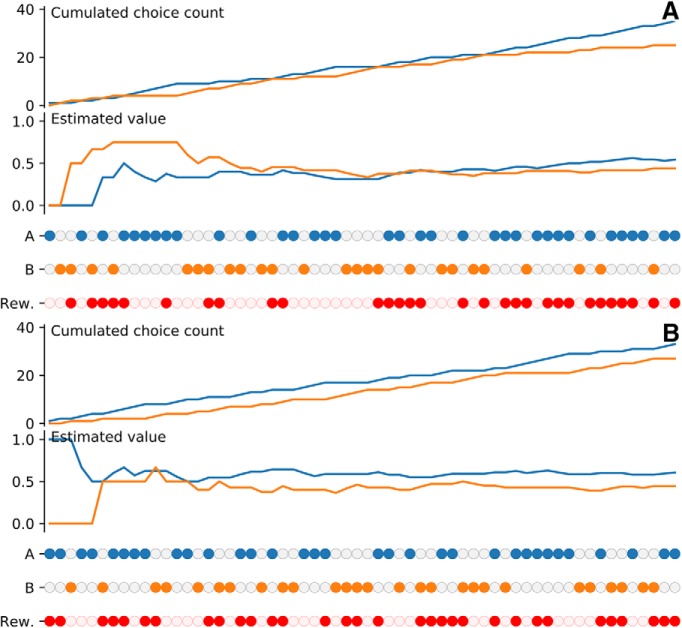
Monkey performance during a single session. Filled dots indicate the chosen cue between A and B. Filled red dots indicate if a reward has been received following the choice. Reward probability is 0.75 for cue A and 0.25 for cue B, but the displayed values are computed according to the actual reward received for each option. They are based on the history of the session, not the theoretical values. ***A***, In saline condition (C0), the monkey is able to slowly choose for the best cue with a slight preferences for A at the end of the 60 trials. Estimation of the perceived value of the two cues shows the actual value of A is greater than the value of B at the end of the session ***B***, In muscimol condition (C1), the monkey chooses cues randomly as indicated by the overall count of choices A and B. Estimation of the perceived value of the two cues (dashed lines) reveals a greater estimation of the value of A compared to the value of B.

This can be simply understood by scrutinizing a session in control and lesion condition (Fig. 8). In control condition, the model learns to select the best cue thanks to the BG. Learning the best stimulus induces a preferential selection of the best stimulus to obtain a higher probability of reward. If the process is repeated over many trials, this leads implicitly to an over- representation of the more valuable stimuli at the cortical level and consequently, Hebbian learning will naturally reinforce this stimulus. In the lesion condition, selection is random and each stimulus is roughly selected with equal probability, which allows the BG to evaluate the two stimuli even more precisely. We believe this is the same for the monkeys although we do not have access to internal values and weights. However, we can see in Figure 9 that the estimated value of stimuli (computed as the probability of reward) reflects the highest value for the best stimulus. Similarly, the number of times a given stimulus has been selected is correlated with its actual value.

### From reinforcement to Hebbian learning

These new results, together with our previous results (Piron et al., 2016), shed light on a plausible neural mechanism responsible for the gradual mix between an A-O and a S-R behavior. The novelty in our hypothesis is that two systems that act and learn together, and we tend to disagree with the hypothesis of a hierarchical system (Dezfouli and Balleine, 2013). In our case, the final behavioral decision results from a subtle balance between the two decisions. When a new task needs to be solved, the BG initially drives the decision because initially it has a faster dynamic. In the meantime, the cortex takes advantage of this driving, and gradually learns the decision independently of the reward. We’ve shown how this could be the case for monkeys, although we lack experimental evidence that the decision in muscimol condition is actually driven by the cortex. The actual combination of the two systems might be more complex than a simple weighted linear combination and this make the study even more difficult to carry on. What we see at the experimental level might be the projection of a more complex phenomenon. Persisting in a devaluated task does not mean that the system is *frozen*, but the time to come back from a S-R oriented behavior might be simply longer than the time to initially acquire the behavior.

10.1523/ENEURO.0339-17.2018.supplement Supplementary 1 Supplementary Model Codes. Download Supplementary 1, ZIP file

10.1523/ENEURO.0339-17.2018.supplement Supplementary 2 Supplementary Experimental data. Download Supplementary 2, ZIP file

## Synthesis

Reviewing Editor: Bradley Postle, University of Wisconsin Decisions are customarily a result of the Reviewing Editor and the peer reviewers coming together and discussing their recommendations until a consensus is reached. When revisions are invited, a fact-based synthesis statement explaining their decision and outlining what is needed to prepare a revision will be listed below. The following reviewer(s) agreed to reveal their identity: Sanjay Manohar, Fred Hamker.

The reviewers have seen each others reviews and, in particular, R1 is in agreement with R2 that the title and abstract, as currently written, do not accurately convey the content of the manuscript.

Reviewer #1:

This manuscript first presents a model of the cortex-basal ganglia interaction. Then behavioural data from two monkeys on a rewarded stimulus-selection learning task is shown, before, during and after muscimol-inactivation of GPi. The authors showed that GPi inactivation makes decisions worse, but does not impair learning. The model exhibits a similar pattern.

This study is nicely conceived and shows how models of the basal ganglia can generate rich behavioural predictions. The model appears up-to-date and plausible. The task is interesting and the behavioural data appears to be of good quality. The study is topical and of fairly broad interest. I liked the way the model was phrased in a general way, then applied to the specific case. It is also really excellent that the authors have included their data.

However the degree to which the model interdigitated with the data was less than I had hoped. There appear to be several crucial predictions that the model makes about the data, which were not adequately tested. My main criticism is that the some analysis detail is lacking. I would like to see the following issues addressed before this can be published:

Major

-----

1. stronger characterisation of model

1.1 There are a number of missing links in the description of applying the model to the data

a) how many parameters in the full model? How were they fitted? Was any of the fitting based on looking at the data / matching the data?

b) Why does the model produce the silent learning effect? Is it i) intrinsic to the model setup, ii) dependent on fitting appropriate parameter values, or iii) an independent prediction?

c) The model is fairly ‘heavyweight’-- why? is it because: i) it is needed to account for the locations changing on each trial, that can't be achieved by simpler RL models. ii) a specific pattern of behaviour that can't be explained in simpler models, iii) to be generalisable to tasks that are not expanded upon in this paper - maybe the authors can give specific examples of existing tasks and datasets that could be tested against this model? iv) It matches biology better?

d) how does the model solve the task? a stepwise walkthrough is needed. Why should the three components (cognitive, associative and motor) be connected together in the way they are? Authors should relate the connections in Fig4 to the functional role that each component plays in the computation of the appropriate action. How is the model is similar / different to its predecessors (esp Frank & OReilly, Redgrave & Gurney, Doya/Hikosaka).

1.2. I would recommend showing some standard learning metrics.

a) stick-vs-switch (Probability of choosing the same stimulus as on the previous trial) on the Y axis, and the previous trial feedback (reward vs penalty) on the x-axis. This could be split up by condition/session, or by early/late trials, or can also be done at various 'history delays' to get a learning falloff curve (eg. P_same_response_as_n-2 vs reward_or_penalty_on_n-2) as required. These effects can presumably be seen in the model too.

b) What would be really useful is to use this kind of plot to quantify ‘perseverative learning’ i.e. learning onto the action domain , rather than onto the stimulus domain. In other words, plot the y-axis with ‘Probability of choosing the same motor action as previous trial’ instead, as a function of previous reward. The model may make interesting predictions about whether there is true stimulus-value or action-value learning:

i) If shapes (A-left) & (B-right) are presented on trial n, (A-left) is selected and wins, then on trial n+1, shape (A-top) & (B-bottom) is presented, is the monkey more likely to choose (A-top) than if it previously lost? [=stimulus-value]

ii) If shapes (A-left) & (B-right) are presented on trial n, (A-left) is selected and wins, then on trial n+1, shape (A-left) & (B-top) is presented, is the monkey more likely to choose (A-left) than if trial n had (A-bottom) & (B-right) - in other words does the monkey have a component of location-repetition learning? [=action-value]

iii) are these two effects dependent on GPi in the data or model?

1.3. It appears that the model didn't do very well on sessions 4 and 5, even with GPi on. This is odd, in fact it appears that the model was consistently significantly below chance in session 4. This pattern is not seen in any of the monkey sessions.

1.4. The model predicts reaction times - these are not mentioned but would fit well in this paper. Do RTs fall across a session and are they affected by the artificial lesion? Is there a difference for error/correct trials?

2. interpretation that silent learning occurs

The benefit at C2 start, compared to C0 start, might not be accounted for by reinforcement learning, but by stimulus familiarity causing faster learning at the very start of C2. Or it could be due to a lower reward expectation, since in C1 monkeys would have received less reward than in C0. Ideally there should be a control condition ‘C1a’ where the same stimuli were presented but with randomised feedback matched to performance in C0, so that everything was the same apart from the opportunity to learn. One way around this lack of control condition, would be to look just at the very first trial of C2 - were they above chance? There is a possibility for one-shot learning in this kind of task, though it depends on the feedback statistics. Further, you could analyse the learning rate at the start of C2. If the benefit is mediated by familiarity alone, then the learning rate should be faster at the start of C2 than at the start of C0; however if the authors are correct that there was learning during C1, then the learning rate at the start of C2 should instead be lower (or at least, not different). I assume this is the case for the model. The authors may also want to test the interaction term between C0_first, C0_last, C2_first, C2_last, which would indicate that there was less learning in C2 than C1.

3. ‘illegal’ ;responses

What proportion of the errors were due to monkeys making an 'illegal' response, vs making the wrong choice? Note that performance is considerably below 50% for some muscimol sessions. This presumably must be due to increased illegal responses? I assume this is not the case at the start of C0, where monkeys are presumably choosing a valid but incorrect response? Colouring the errors in a different way on the circles of fig.15 might be a useful way to visualise this - maybe even missing out the circle altogether for illegals?

NOTE:

It is excellent that the authors have provided their model code. In particular I must note that the code was exceptionally well presented and clear to follow.

Minor

------

1. modelled value

Figure 9: It was unclear where ‘Weight’ and ‘Value’ are calculated from in the model. The sentences are v confusing - the value of B should be 0.75 not 0.25, shouldn't it?

Fig 10 / line363 -‘estimation of the perceived value of the two cues’ - how was this done?

12.243: I could not see the explanation of the calculation of V and RPE. Where in the brain might V be represented? Is PE the same as RPE?

Regarding these, it would make sense to show a 'binned plot' showing the proportion of correct responses as a function of the model's estimated value. This would demonstrate that the modelled value predicts choice. Can a similar plot also be made for specific action values - i.e. can the model also predict the type of error monkeys make eg to the wrong shape or to invalid locations?

2. English

Several dozen grammatical errors are present throughout the article. I do not know what the authors' language is, but I would recommend that a native english speaker should thoroughly read and advise. The errors are mainly on plurals and articles.

Just a few examples are

204 ‘dyanmic’

10.211 ‘all the population’

10.222 ‘cortex cognitive’

line 364 ‘number of time’

p17 ‘choose for the best cue with a slight preferences’

Minor clarifications required

------------------------

3.97: ‘only motor choice is taken into account when computing the reward’ - are you talking about the monkey computing reward, or the task's determination of reward?

7.167 Equation for f(x): assume the exponential should be superscript rather than subscript

the ‘cognitive’ ‘motor’ and ‘associative’ assemblies are not introduced, and it is not clear what the difference between them is in terms of what they represent in the task. Towards the end of the paper it became clear that ‘cognitive’ represents the shapes on screen, and ‘motor’ represents their location.

14.313 It was not clear that what values went into the statistical analysis in table 5. I am assuming it was the proportion of correct choices in each simulated session?

14.314 does ‘Day 1’ correspond to ‘C1’?

It is not made clear (in the figures) that sessions 1-7 and 8-12 were monkeys 1 and 2 respectively. In fact, I am still not sure, was this the case?

The description 357-359 about implicit learning and ‘converting to the temporal domain’ remained opaque to me. ‘Implicit’ usually means without awareness? Can the authors expand on this? Also ‘even if it is not significant’ - what does this mean, significant according to a statistical test, or meaningful, or something else?

line 383: last sentences were very hard to understand. What does it mean? it may need a paragraph of its own.

Fig 7: x axis - no zero.

3.106 ‘legal’ not defined until line 109

Reviewer #2:

The authors provide a monkey study and a computational model in which they show that when GPi is blocked learning on a task is still possible though the selected actions are at random.

My first concern is that the title and parts of the introduction and conclusion are inappropriate. None of the studies reported deals with the problems of learning from S-O to S-R, e.g. by selective devaluation procedures. Thus, I suggest to remove all these aspects, including a change in title, and focus on how the model may explain the experimental observations. I see that the proposed model may be further elaborated for this purpose, but at present the experiment simulated would not classify to particularly relate to S-O or the transition to S-R.

The authors propose a model of BG - cortex interaction, which is composed of at least two loops. The associative loop may be regarded as a third one, but projects only into the other two loops.

From the anatomical point of view, this architecture has not been suggested before. However, it does not seem to be very well supported by data. The model includes connections from the associative striatum to the GPi of both the cognitive and motor loop. Many previous studies on the connectivity of the basal ganglia have shown that the associative section of the striatum is mainly connected to its own group of pallidal cells. The authors should therefore show evidence of the existence of the connection to multiple different GPi areas or at least acknowledge that this is an assumption not based on data and motivate the model by a proposed function or any other reason.

See the following references for the standard known connectivity:

-K. Nakano, T. Kayahara, T. Tsutsumi & H. Ushiro (2000) Neural circuits and functional organization of the striatum. J NEurol

-C. Francois, J. Yelnik, G. Percheron and G Fenelon (1994) Topographic distribution of the axonal endings from the sensorimotor and associative striatum in the macaque pallidum and substantia nigrsa. Exp Brain Res.

-S. N. Haber. (2003) The primate basal ganglia: parallel and integrative networks. Journal of Chemical Neuroanatomy.

Let us now focus on the prediction tested by the experiment. On line 300 the authors claim that their model predicts that covert learning of stimulus value can occur even if the BG output is blocked and as a result the behavioral choice is initially random. However, the only requirement for making this prediction is that the striatum is informed by the final choice. There may be several different solutions to how this transfer is realized, but this prediction is inherent to several previously published models. Some examples are:

-Brown, J.W., Bullock, D. & Grossberg, S. (2004) How laminar frontal cortex

and basal ganglia circuits interact to control planned and reactive saccades. Neural Networks -Frank, M. J., Samanta, J., Moustafa, A. A., and Sherman, S. J. (2007). Hold your horses: impulsivity, deep brain stimulation, and medication in parkinsonism. Science

-Schroll, H., Vitay, J., Hamker, F.H. (2014) Dysfunctional and Compensatory Synaptic Plasticity in Parkinson's Disease. European Journal of Neuroscience

Thus, the prediction itself is not particularly novel, but still interesting as it has not been proposed and tested before.

First: The authors should provide credit to those models that would make a very similar prediction to the one of the authors.

Second: The exact mechanisms of how this model achieves the task is difficult to understand in the present manuscript and should be improved.

- In particular, there are several pathways from cognitive CTX to the motor loop. Which of those pathways is doing what?

- Learning takes place at two sites. Explain more what is really learned there.

- What happens when the cortical pathway contradicts the basal ganglia pathway? Who controls who? What is the arbitration mechanism?

- Regarding learning in the cortico-cortical connections: aren't these connections just learning a copy of the fixed connections from the cognitive cortex to the associative striatum.

- The model suffers from a binding problem. Obviously the authors use a ‘trick’ to solve the problem (see from line 194 ongoing). Without this trick, the model cannot even learn this simple task. I personally find this very problematic. Please specify in Fig. 4 the inputs into the model and how you solve the binding problem.

Fig. 9 and 10 are not very intuitive. The reward probability of cue A is larger but B is more often chosen in the intact model?

Fig.9 and 10: How is the value in model and monkey computed? Put those figures into the results, not discussion.

Other comments:

The description of the connectivity patterns in Table 3 is not clear enough to fully understand the structure of the model, e.g. what is the meaning of (i,1)->(i,*)?

The learning rule for the cortico-striatal synapses does not consider pre-synaptic activity, only postsynaptic. The authors should comment on this.

Line 135: The motivation to exclude the indirect pathway is very simplistic and should be improved.

Fig. 3: Remove the GPe, as it is not part of the model.

Line 174 ongoing: Several observations are described but not how exactly they are considered in the model? After each observation, specify how you translate this into the model.

Line 191: What is the reason for the higher gains to the cognitive and motor parts?

Line 236 describes a parameter mu which correspond to the actor learning rate. However, this parameter does not appear in equation 3 and 4 where it is supposed to be.

Line 243: What is PE?

On line 314 the authors mention that they used only 12 different simulations. This is a very low number considering that neuron equations have a noise term. Thus, the error of the model evaluation is of the order of 1/sqrt(N), with N the number of samples (or simulations). The authors should perform a larger number of simulations in order to show that they results are not an effect of stochasticity.

On line 341 the authors state that their results reinforce the idea that the basal ganglia is based on an actor-critic architecture. This is not completely clear. The authors should indicate what sections of the model correspond to the actor and which to the critic. The following lines suggest that the BG is the critic, however, according to Figure 4 it is capable of selecting actions, converting it into the actor.

Line 367: The authors claim that the results shed a new light on a plausible neural mechanism responsible for the gradual mix between an action-outcome behavior and a stimulus response one. The authors however never show a numerical experiment in which this happens. Such experiments have shown that animals tend to initially have a goal directed behavior and then develop habitual responses. However this is not shown on any of the simulations here. In order to correctly claim this, the authors should simulate a devaluation protocol, as those usually used in biological experiments to test the changes between the two types of behavior. Alternatively, as suggested above, these claims can be dropped.

Line 370: The authors say they disagree with the hypothesis of a hierarchical system. However, I believe their basal ganglia model is hierarchical. In the competing parallel model both loops represent completely independent learning systems. The author's model is clearly not parallel. In a hierarchical model the first loop makes a selection, which is then passed to the next loop which executes the action as in the model presented in the paper. The only special thing in the author's model is the inclusion of a cortical pathway that bypasses the two loops.

Fig. 8: Comment on the learning in the model. Is this really so slow or does it occur due to averaging? How do individual learning curves look like?
